# The Secreted Aminopeptidase of *Pseudomonas aeruginosa* (PaAP)

**DOI:** 10.3390/ijms25158444

**Published:** 2024-08-02

**Authors:** Efrat Kessler

**Affiliations:** Maurice and Gabriela Goldschleger Eye Research Institute, Faculty of Medicine and Health Sciences, Sheba Medical Center, Tel Aviv University, Ramat-Gan 5262000, Israel; ekessler@tauex.tau.ac.il

**Keywords:** *Pseudomonas aeruginosa*, extracellular proteases, proteolytic processing, aminopeptidase, protease inhibitors, virulence, pathogenicity

## Abstract

*Pseudomonas aeruginosa* is an opportunistic pathogen that causes severe infections in compromised hosts. *P. aeruginosa* infections are difficult to treat because of the inherent ability of the bacteria to develop antibiotic resistance, secrete a variety of virulence factors, and form biofilms. The secreted aminopeptidase (PaAP) is an emerging virulence factor, key in providing essential low molecular weight nutrients and a cardinal modulator of biofilm development. PaAP is therefore a new potential target for therapy of *P. aeruginosa* infections. The present review summarizes the current knowledge of PaAP, with special emphasis on its biochemical and enzymatic properties, activation mechanism, biological roles, regulation, and structure. Recently developed specific inhibitors and their potential as adjuncts in the treatment of *P. aeruginosa* infections are also described.

## 1. Introduction

*Pseudomonas aeruginosa* is a ubiquitous environmental bacterium, also known as an important opportunistic pathogen, which can cause acute and chronic infections in compromised hosts such as burn victims, cancer patients, and cystic fibrosis (CF) patients [1,2]. *P. aeruginosa* infections are difficult to treat because of the ability of the bacteria to develop drug resistance, secrete many virulence factors, and form biofilms [3,4,5,6]. Virulence factors secreted by *P. aeruginosa* include enzymes such as phospholipases, lipases and proteases, toxins (exotoxins A, T, and S), pigments (pyocyanin, pyoverdine), rhamnolipids, and alginate [3,4,5,6].

Proteases are hydrolytic enzymes that cleave peptide bonds in proteins and peptides. Based on the location of cleavage within the protein/peptide substrate, proteases are classified into endo- and exopeptidases. The endopeptidases cleave internal peptide bonds, whereas the exopeptidases cleave terminal peptide bonds, releasing single amino acids, dipeptides, or tripeptides from either the C- or the N-terminal end of the substrate. Accordingly, they are termed carboxy- and aminopeptidases [7]. Based on their catalytic mechanism, proteases are classified into aspartic, cysteine, metallo-, serine, and threonine proteases, as well as a group of proteases with an unknown mechanism [7,8].

*Pseudomonas aeruginosa* secretes several endopeptidases, including elastase (pseudolysin, LasB), alkaline proteinase (aeruginolysin, Apr), LasA protease (staphylolysin, staphylolytic endopeptidase), and a lysine-specific endopeptidase (protease IV, LysC, PrpL) [5,6,9,10]. Elastase is the most abundant and most potent endopeptidase secreted by *P. aeruginosa,* unique in its ability to degrade elastin. It preferentially cleaves peptide bonds on the amino side of hydrophobic amino acid residues (Phe, Leu), and can degrade many host proteins in addition to elastin, including collagens, proteoglycans, immunoglobulins, complement components, cytokines, surfactant proteins A and D (components of the lung innate immune system), and more. Elastase is therefore a major contributor to the pathogenesis of *P. aeruginosa* infections [11,12]. Although far less potent than elastase, Apr can degrade a variety of biologically important host proteins, including laminin, cytokines, and immunoglobulins, thus also contributing to the pathogenesis of *P. aeruginosa* infections [13]. LasA protease cleaves peptide bonds on the carboxyl side of Gly-Gly pairs in proteins, which are common within the peptidoglycan network of *Staphylococcus aureus* and are also present in elastin. Cleavage by LasA protease of such peptide bonds within the cell wall peptidoglycan of *S. aureus* causes lysis and killing of the bacteria. By nicking elastin, LasA protease increases the elastinolytic potential of elastase dramatically, enhancing damage to elastic tissues such as the lungs and blood vessels [14]. LysC cleaves exclusively peptide bonds on the carboxyl side of lysine residues in proteins and peptides [15]. Unlike elastase, LasA protease, and Apr, which are all Zn-dependent metalloproteases, LysC is a serine protease, strongly inhibited by the lysine-specific serine protease inhibitor Tosyl-lysyl-chloromethyl-ketone (TLCK). LysC cleaves fibrinogen [15] and inactivates surfactant proteins A, D, and B, thus interfering with clot formation and innate immune defense mechanisms [16].

In addition to the endopeptidases, *P. aeruginosa* secretes an aminopeptidase that removes single amino acids from the N-terminal end of proteins and peptides designated PaAP (*P. aeruginosa* aminopeptidase) [17]. PaAP is thought to complement the activity of the endopeptidases, producing low molecular weight nutrients that are consumed by the bacterial cells, thus promoting bacterial proliferation and invasiveness. The secreted proteases of *P. aeruginosa* combined can cause severe damage to host tissues and facilitate bacterial evasion of the host’s innate immune system.

Elastase, LasA protease, LysC endopeptidase, and PaAP are secreted via the type 2 secretion system (T2SS) that, in *P. aeruginosa,* consists of 11 Xcp (extracellular protein) proteins, whereas Apr is secreted via a type 1 secretion system (T1SS) that is specific for Apr [6,18]. Expression of most virulence factors of *P. aeruginosa*, including many of the proteases and the Xcp proteins of the T2SS, is regulated by several cell-density-dependent regulatory systems called quorum sensing (QS) [19].

PaAP is abundant in the biofilm matrix [20] and has been shown to modulate biofilm development [21,22]. It is also a major component of *P. aeruginosa* outer membrane vesicles (OMVs), especially OMVs from clinical isolates, which are implicated in virulence and nutrient acquisition [22]. This further highlights PaAP as an important virulence factor of *P. aeruginosa*. The present review summarizes the current knowledge on PaAP, emphasizing aspects such as structure, proteolytic activation, biochemical and enzymatic properties, regulation, and role in pathogenesis. The potential of PaAP as an emerging therapeutic target is also discussed.

## 2. History and Names

The occurrence of a secreted aminopeptidase in *P. aeruginosa* was initially predicted in 1998 by Braun et al. [23] who detected an unknown 58 kDa protein in the culture medium of a *P. aeruginosa* mutant lacking elastase and Apr, which showed high sequence homology with the secreted aminopeptidase of *Streptomyces griseus*. Their prediction was confirmed in 2001 by Cahan et al. [17], who demonstrated that a 56 kDa protein encoded by the same gene as the putative aminopeptidase possesses aminopeptidase activity. The name PaAP given by Cahan et al. [17] matched previously established terminology for microbial aminopeptidases, including *S. griseus* aminopeptidase (SGAP) [24], *Bacillus subtilis* aminopeptidase (BSAP) [25], and *Aeromonas proteolytica* aminopeptidase (AAP) [26]. Because hydrolysis by PaAP of the classical aminopeptidase substrate Leu-p-nitroanilide (Leu-pNA) was far more efficient than hydrolysis of several other amino-acid p-nitroanilide derivatives, PaAP was also termed leucine aminopeptidase (LAP) [17]. The name PaAP has been adopted by most investigators [21,22,27,28,29,30], although the names PaAP and LAP have also been used interchangeably [22], and the name LAP has also been modified to PA-LAP (*Pseudomonas aeruginosa* LAP) [31]. Subsequent studies on PaAP revealed that Lys-pNA is in fact cleaved far more efficiently than Leu-pNA [32,33], so it was also termed lysine aminopeptidase. PaAP derived from the solvent-stable *P. aeruginosa* strain, pseA, was called pseA aminopeptidase [34]. The encoding gene, *pepB*, and its chromosome number, PA2939, have also been used as names, especially in studies involving proteome/transcriptome analyses of proteins secreted by *P. aeruginosa* [20,35]. Out of the various names, PaAP is the most appropriate because it follows an existing terminology and does not relate to the properties of the enzyme.

## 3. Activity, Specificity, and Basic Properties

Identification of a protease as an aminopeptidase is commonly based on its ability to cleave synthetic substrates consisting of an amino acid coupled to a chromogenic or fluorogenic group via an amino-acyl bond. Leu-p-nitroanilide (Leu-pNA) is the standard chromogenic substrate widely used to determine the activity of broad-spectrum aminopeptidases [36]. Leu-pNA was therefore the substrate of choice for PaAP identification and activity determination in most of the studies [17,29,30,31,32,33,34]. PaAP also hydrolyzes the fluorogenic substrate leucine-7-amido-4-methylcoumarin (Leu-AMC), offering a highly sensitive method for the determination of its activity [9,33]. Another, fluorogenic substrate for determination of PaAP activity contains resorufin as the fluorescent group, coupled to leucine via p-aminobenzyl alcohol as a quencher. Removal of leucine by PaAP leads to an increase in fluorescence due to the release of free resorufin [37]. This fluorometric assay is also highly sensitive.

The cleavage specificity of PaAP has been studied by several groups, all using various amino acid-pNA derivatives as substrates [30,32,33,34,38,39]. The results were not fully consistent, perhaps because the assay conditions differed from one laboratory to another (different pHs, buffers, temperature, etc.). Nonetheless, combined, the results agreed that Leu-pNA is readily cleaved and can be used as the standard substrate for the determination of PaAP activity (Table 1, and each of the above citations). Cleavage of other aliphatic amino acid residues such as Ile, Val, Ala, and Met is far less efficient, and Phe-pNA is a very poor substrate, cleaved at least 100-fold less efficiently than Leu-pNA [30]. Arg-pNA is also a poor substrate (Table 1A and ref. [30]). Most striking, Lys-pNA was found to be cleaved 2–3-fold more rapidly than Leu-pNA and also more rapidly than all of the other substrates tested (Table 1) and [32,33]. In one instance [30], Lys-pNA was cleaved about 2-fold more slowly than Leu-pNA, yet, it was cleaved more efficiently than all of the other substrates tested (Table 1A). It can be concluded that PaAP has a relatively broad substrate specificity.

PaAP can process peptides of various lengths (dipeptides to peptides containing 25 amino acid residues) [30]. These include a casein-derived peptide, RYLGYL, a peptide having the same sequence as its own C-terminus, ERWGHDFIK, and a peptide corresponding to its own N-terminus, SEAQQFTEFW, which is unstructured. Complex peptides containing secondary structures and disulfide bonds such as human defensins, and N-blocked peptides like Ac- ERWGHDFIK [30], Z-Leu-Ala, and N-acetyl-Ala_4_ [17], are not cleaved. Leu-Ala and Ala_4_, having free N-termini, are both cleaved readily [17]. Time course studies [30] indicated that some peptides are cleaved in a processive manner, i.e., the cleaved product remains bound to the enzyme for further cleavages, while other peptides are cleaved distributively, i.e., the first cleavage product dissociates from the enzyme, followed by re-binding and cleavage of the new N-terminal residue, and so forth. It was suggested that in fact, the processing of peptides is mixed; the first few residues are cleaved processively, while subsequent amino acids are removed in a distributive manner [30]. PaAP can also hydrolyze pesticides such as β-Cypermethrin [38]. This, however, is puzzling because β-Cypermethrin and its analogs have no free amino group nor do they contain an amino acid residue.

PaAP is heat stable, retaining its activity at 60–70 °C [17,32,38,39], and its optimal temperature for activity is also high, 60 to 75 °C [32,38,39]. The optimal pH for activity is 8.0–9.0 [17,32,34,38,39]. PaAP is stable to organic solvents, including DMSO, methanol, ethanol, acetone, hexane, heptane, etc. [34,39]. PaAP is inhibited by metal chelators, including 1,10-phenanthroline, tetraethylene pentamine, EDTA, and EGTA, but not by serine protease inhibitors such as PMSF, DFP, DCI, or inhibitors of cysteine proteases such as NEM [17]. Cysteine-modifying agents such as p-chloromercuric benzoate (pCMB) also do not affect PaAP’s activity [34]. Reducing agents such as DTT and β-mercaptoethanol fully inhibit PaAP’s activity [17,39]. The effect of metal ions on PaAP’s activity was tested by several groups [32,38,39]. The only metal ions showing significant inhibition (>60%; 1 mM) include Cu^+2^, Hg^+2^, Ni^+2^, and Zn^+2^ (at 1 mM). Co^+2^ increased PaAP’s activity up to 7 fold, depending on its concentration [32,38].

Amastatin, a common inhibitor of aminopeptidases, and Balsalazide, an FDA-approved anti-inflammatory drug, inhibit PaAP’s activity at the µM concentration range, and both inhibit biofilm formation by *P. aeruginosa* [37]. Cyclic-ERWGHDFIK, a peptide corresponding to the C-terminal tail of the inactive precursor of PaAP, cyclized via an isopeptide bond between the glutamate and lysine side chains, is a highly potent inhibitor of PaAP with a K_i_ of 22.8 nM [30]. It inhibits Leu-pNA hydrolysis by PaAP almost completely at 500 nM and diminishes substantially biofilm formation by *P. aeruginosa* at 100 µM [30].

## 4. Structural Chemistry

**Figure 1:**
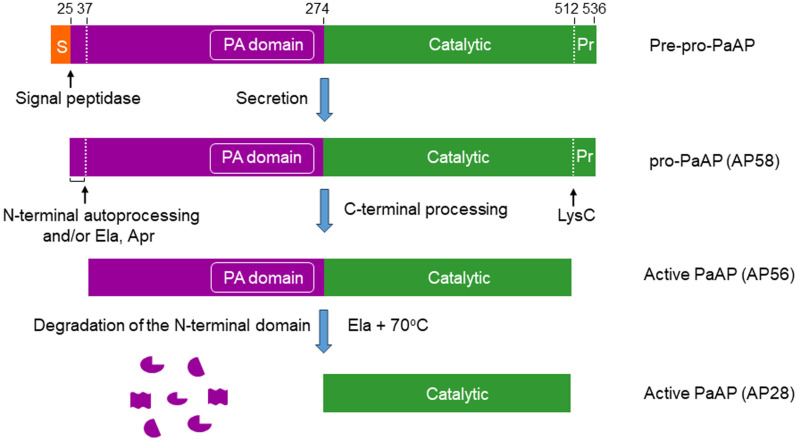
Domain structure and proteolytic processing of pro-PaAP. PaAP is produced as a preproenzyme. The signal peptide is removed by a signal peptidase upon passage through the inner membrane. PaAP is secreted as an inactive proenzyme (pro-PaAP, AP58), with an apparent molecular weight of 58 kDa [33]. Activation requires proteolytic removal of a short C-terminal propeptide (residues 513–536) by LysC [31,33] and is followed by autoproteolytic processing of a short N-terminal sequence (residues 25–36) [31]. Elastase and/or Apr may also be involved in this step [17,33]. The active form of PaAP (apparent molecular weight, 56 kDa) is also known as AP56 [17,33]. In vitro, the N-terminal inactive domain of AP56 can be degraded by elastase at 70 °C, generating the free catalytic domain (AP28; apparent molecular weight, 28 kDa) [17]. S, signal peptide; Pr, propeptide; PA, protease associated domain.

PaAP is produced as a pre-proenzyme containing 536 amino acid residues [17] and a mass of 57,511 Da. The signal peptide (pre-peptide) is 24 residues in length, followed by an inactive N-terminal domain (positions 25–273) that contains a protease-associated (PA) domain [40,41] (positions 117–272). The inactive N-terminal domain is followed by the catalytic domain (239 residues), and a 24-residue C-terminal pro-peptide (Figure 1) that retains the pro-enzyme in an inactive form.

**Figure 2:**
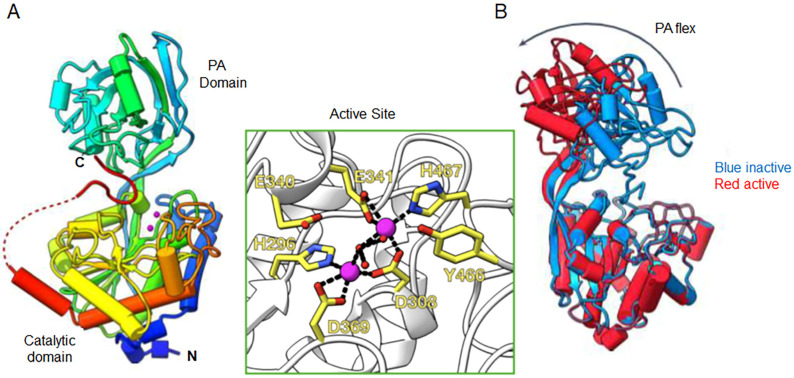
Three-dimensional structure of PaAP. (**A**), Structure of full-length pro-PaAP solved at 1.4 Å. The secondary structure is rainbow-colored, from the blue N-terminus to the red C-terminus. The pink spheres are zinc ions that indicate the location of the active site. A short region of disorder between the C-terminus and the peptidase domain is shown by a red dashed line. Cleavage by LysC (and elastase) occurs within this unstructured region. The green box is an enlargement of the active site, highlighting Asp308, Asp 369, His296, Glu341, and His467, the residues involved in the coordination of the two zinc atoms (magenta spheres). Glu340 and Tyr 466, which complete the active site, are also indicated. (**B**), Comparison of the structures of pro-PaAP (inactive, blue) and the active form of PaAP (red). A conformational change of the PA domain is evident in the active enzyme, which allows access of substrates to the active site cleft. From Harding et al. [30], with slight modifications. The PDB numbers of the structures of the PaAP variants reported [30] are 8ACR, 8ACK, 8AC7, 8AC9, and 8ACG.

The 3D structure of full-length pro-PaAP shows that the C-terminus (IERWGHDFIK; residues 527–536) is folded with a hairpin turn and is separated from the catalytic domain by an unstructured stretch of 16 amino acid residues: QKAQSRSLQMQKSASQ (residues 511–526) [30]. The structured C-terminus (IERWGHDFIK) binds in the active site cleft between the PA and the peptidase domains (Figure 2A) [30]. The C-terminal free carboxyl group interacts with the positively charged side chain of Arg194, and the side chains of His532 and Asp533 of the β-hairpin coordinate a water molecule and are positioned directly above the active site pocket. Together, these interactions block access of the substrate to the active site [30]. The catalytic domain is composed of an eight-stranded β-sheet surrounded by helices. The mixed α/β PA domain is attached to the catalytic domain via an extended β-strand. There are three disulfide bonds, two in the peptidase domain, towards the N- and C-termini, and one in the PA domain [30]. The cysteine residues involved in each S-S bond are not specified.

The structure of the active enzyme, lacking the C-terminus, reveals a major conformational change compared to that of the full-length pro-PaAP [30]. Removal of the inhibitory C-terminus leads to a −40° rotational transition of the PA domain in comparison to the full-length PaAP (20 Å translation at its furthest point). This conformational change increases the opening between the PA and peptidase domain, creating a more accessible active site (Figure 2B) [30].

PaAP belongs to the M28 family of co-catalytic metalloproteases, and its catalytic domain shows high sequence homology to other family members, in particular, *Streptomyces griseus* aminopeptidase (SgAP; 52% identity) [17]. Based on sequence identity, it was predicted that PaAP contains two zinc atoms coordinated by Asp308, His296, Asp 369, Glu341, and His467, which are required for catalytic activity [17]. Consistently, recombinant PaAP mutants D308A and D369A are inactive [31]. The 3D structure of PaAP (Figure 2A) [30] confirms these predictions, showing that Zn1 is coordinated by Asp308, His296, and Asp 369, while Zn2 is coordinated by Glu341, His467, and Asp308 (which coordinates the binding of both zinc atoms) (Figure 2A, green box) [30]. Coordinated between both Zn atoms is a water molecule, whose activation to OH^-^ is crucial for catalysis. Residues Glu340 and Tyr 466 complete the active site.

## 5. Secretion, Maturation, and Regulation

PaAP is secreted via the type II secretion pathway, which includes two steps for secretion across the inner and outer membrane [18]. After removal of the signal peptide upon passage through the inner membrane (step 1), the resulting pro-PaAP is secreted across the outer membrane via the Xcp export machinery (step 2) [23] and is activated extracellularly (Figure 1) and [33]. Activation requires C-terminal processing that is mediated by LysC cleavage of the peptide bond between Lys512 and Ala513, close to the C-terminal end of pro-PaAP [30,31,33]. Elastase can also lead to PaAP’s activation by cleavage of the peptide bond Ser517-Leu518 downstream of the LysC cleavage site [31]. Both cleavages occur within the unstructured linker that separates the structured C-terminus and the catalytic domain (Figure 2) and is apparently sensitive to proteolysis. Ela and Apr can both contribute to PaAP’s activation indirectly via proteolytic activation of pro-LysC [33,42]. C-terminal processing of pro-PaAP is followed/accompanied by limited N-terminal processing either autocatalytically and/or by Ela/Apr, leading to the removal of the first 12 amino acid residues. The N-terminal residue in active PaAP is Thr37 (Figure 1) and [30,31,33].

**Figure 3:**
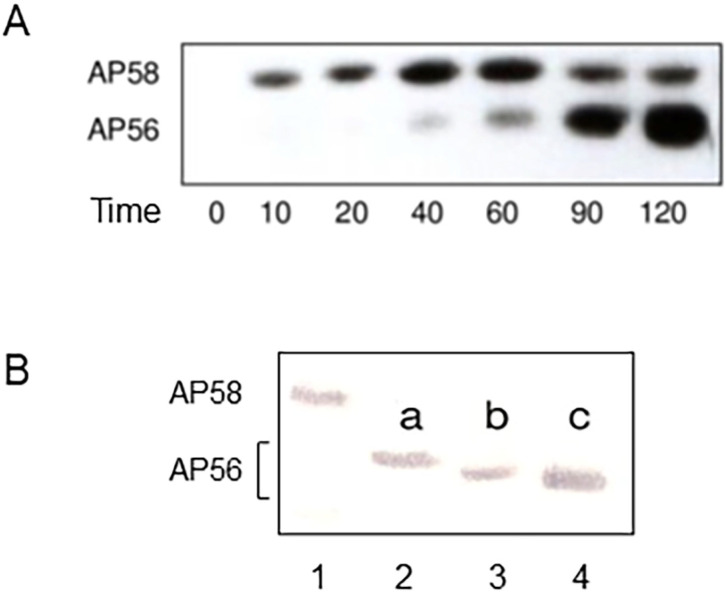
PaAP is secreted as an inactive proenzyme (AP58) that is processed extracellularly to the active enzyme (AP56). (**A**), Immunoblot showing that PaAP is secreted as a 58 kDa protein and is converted after secretion to a 56 kDa protein. Time, minutes after secretion. (**B**), Immunoblot demonstrating three different forms of processed PaAP, AP56a, AP56b, and AP56c. Lane 1, pro-PaAP (AP58) detected in a freshly collected culture filtrate of a ΔElaΔApr *P. aeruginosa* mutant strain; lane 2, AP56a detected in the concentrated medium of the same ΔElaΔApr mutant strain, a product of LysC cleavage; lane 3, AP56b, detected in a *P. aeruginosa* strain lacking Ela but expressing Apr and LysC; lane 4, AP56c detected in the culture filtrate of a wild-type *P. aeruginosa* strain. All three forms of AP56 are equally active [33]. Modified from Axelrad et al. [33].

The secreted inactive pro-PaAP (AP58) migrates in SDS-gels as a 58 kDa protein (Figure 3 and refs. [23,33]), although its calculated molecular weight is 55.1 kDa [31]. Depending on the degree of processing, three distinct protein bands of ~56 kDa, all representing active forms of PaAP, have been detected in SDS-gels (Figure 3B), designated AP56a, AP56b, and AP56c [33]. AP56a is the product of C-terminal processing by LysC, while AP56b and AP56c, which are smaller than AP56a, reflect different N-terminal processing intermediates (Figure 3B and ref. [33]). In vitro, heating of AP56 at 70 °C in the presence of elastase leads to degradation of the entire inactive N-terminal domain, releasing the catalytic domain, designated AP28 (Figure 1); its N-terminal residue is Thr273 [17,33]. Kinetic constants for hydrolysis of several amino acid–pNA derivatives by AP56 and AP28 are essentially identical [33], suggesting that the PA domain has no effect on the hydrolysis of low molecular weight substrates [33].

PaAP differs from the closely related aminopeptidases, SGAP and AAP, in that under physiological conditions both are found in their respective culture filtrates as proteins of about 30 kDa, encompassing purely the catalytic domain [24,26]. This could be related to the presence in PaAP but not in AAP (and probably also SGAP) of the PA domain. The PA domain in PaAP may not only be involved in the maintenance of pro-PaAP as an inactive precursor but may also be involved in additional features of mature PaAP, such as mediation of binding to protein substrates, facilitating their cleavage by PaAP, or interactions with OMV components required for PaAP’s incorporation into the vesicles.

There are two acyl-homoserine lactone (acyl-HSL) quorum sensing systems in *P. aeruginosa*, LasI-LasR and RhlI-RhlR [19]. LasI is responsible for the synthesis of the autoinducer N-3-oxododecanoyl-homoserine lactone (3OC12-HSL), and LasR is a 3OC12-HSL-responsive transcriptional activator. RhlI is responsible for the synthesis of N-butanoyl-HSL (C4-HSL), and RhlR is a C4-HSL-responsive transcriptional activator. PaAP (PA2939) is transcriptionally regulated by the LasR/LasI, but not the RhlR/RhlI, system [35]. Consistently, the addition of 3OC12-HSL but not C4-HSL to the culture medium of a *P. aeruginosa lasI rhlI* double mutant increases the expression of PA2939 [43]. PA2939 transcription is also regulated by the sigma factor RpoS [44]. Expression of PA2939 in the wild-type strain PAO1 is 140-fold higher than in the respective *RpoS* deletion mutant, and 27-fold higher than in a *LasR rhlR* double mutant, indicating that RpoS is the principal transcriptional activator of PA2939 gene expression, more so than the LasI/LasR quorum-sensing system. At the protein level, PaAP’s activity is controlled by post-translational proteolytic processing by LysC and other secreted proteases, as described above.

## 6. Biological and Pathophysiological Aspects

### 6.1. Role of PaAP in Biofilm Development

Biofilms are bacterial communities that adhere to surfaces and are embedded in a self-secreted extracellular polymeric substance (EPS) that confers increased tolerance to environmental stresses and resistance to antibiotics [45]. The capacity to establish biofilms is therefore considered a key virulence factor for a wide range of microorganisms, including *P. aeruginosa*. The main components of the EPS in most microorganisms are polysaccharides, proteins, nucleic acids, and lipids [45]. The biofilm produced by *P. aeruginosa* contains three polysaccharides, alginate, Psl, and Pel, with Psl and Pel present in all strains, and alginate found in mucoid *P. aeruginosa* isolates [46]. PaAP is one of the most abundant proteins in *P. aeruginosa* biofilm [20]. The biofilm of *P. aeruginosa* also contains large amounts of outer membrane vesicles (OMVs), and high levels of PaAP have been found in biofilm-derived OMVs [20]. Together, these suggested a role for PaAP as a modulator of biofilm development in *P. aeruginosa*, which was verified experimentally using both laboratory strains and clinical isolates of *P. aeruginosa*.

#### 6.1.1. Studies with *P. aeruginosa* Strain PAO1

When a ΔPaAP mutant of *P. aeruginosa* strain PAO1 (a laboratory strain) is grown in a minimal medium, a lack of PaAP results in increased cell attachment and biofilm formation at the early stages of biofilm development [21]. After 24 h of growth in such a medium, however, the absence of PaAP leads to massive cell death and biofilm disruption, which in turn, leads to degradation of the Psl matrix by glycosyl hydrolase PslG released from dead cells. Cell death resulted from a lack of nutrients in the absence of PaAP. It was concluded that at the late stages of biofilm development, PaAP is required for nutrient recycling and maintenance of the biofilm matrix. High expression of PaAP is induced at the late stages of biofilm formation by the cell-density quorum-sensing system LasI/LasR.

#### 6.1.2. Studies with Clinical *P. aeruginosa* Isolates

In a lung epithelial cell/bacterial biofilm co-culture model and a ΔPaAP mutant derived from a clinical *P. aeruginosa* isolate, the PaAP mutant formed biofilms with substantially greater cellular biomass and cellular organization during early biofilm development compared to the parental strain [22]. Micro colonies formed by the wild-type strain produced higher amounts of Psl than the mutant strain, indicating that PaAP-dependent modulation of the biofilm matrix can result in an overall more robust biofilm structure. Biofilm detachment activity at the late stages of biofilm development in this model resided in OMVs from wild-type cells but not OMVs from ΔPaAP cells. Furthermore, OMVs from wild-type *P. aeruginosa* exhibited higher proteolytic activity than ΔPaAP OMVs, and cell detachment mediated by wild-type OMVs was sensitive to protease inhibitors. This indicated that OMV-associated proteolytic activity was involved. Increased proteolytic activity in OMVs from wild-type *P. aeruginosa* was dependent on PaAP expression, meaning that when grown in a rich culture medium, as is the case in vivo, PaAP is key to cell dispersion at the late stages of biofilm development in *P. aeruginosa*.

Despite the different models of biofilm formation and growth conditions, the results of both studies indicate that PaAP may function as a modulator of biofilm development.

### 6.2. Role of PaAP in Nutrition

Several authors have suggested that PaAP may play a role in nutrient acquisition [17,20,21]. Their rationale was that PaAP can release free amino acids from protein fragments produced by bacterial endopeptidases, and free amino acids released by PaAP can be taken up by the bacteria, acting as valuable nutrients. Zhao et al. [21] showed that biofilm biomass and planktonic growth of a ΔPaAP *P. aeruginosa* in a minimal medium can both be restored to the level of the wild-type strain when the culture medium is replaced (replenished) every 12 h. This indicated that PaAP can recycle nutrients needed for bacterial growth and proliferation.

Proteome analyses of *rpoS* loss-of-function mutants grown in a protein-based medium (that requires extracellular proteolysis for bacterial growth) revealed that some of the isolated mutants behaved as social cheaters, with low fitness in isolation but high fitness in a mixed culture with the cooperating wild-type strain [27]. Evidently, the *rpoS* mutants exploited an RpoS-controlled public good produced by the wild type but not by the mutant cells, and this public good was identified as PaAP. RpoS is required for the maximal expression of PaAP but has no influence on the expression of the endopeptidases. PaAP apparently complements the activity of the endopeptidases secreted by both the *rpoS* mutant cells and the wild-type cells, thereby providing low molecular weight nutrients needed for the survival of the mutant cells. PaAP therefore seems to be an integral part of a proteolytic sequence in *P. aeruginosa* that permits utilization of proteins as a source of nutrients [27].

### 6.3. PaAP as a Therapeutic Target

In the face of the constantly growing problem of antimicrobial resistance, alternative strategies to combat *P. aeruginosa* infections are needed. Optional therapeutic targets that have been examined to date include extracellular, intracellular, and cell surface virulence factors such as elastase (LasB), quorum-sensing systems, metabolic pathways, biofilms, outer membrane components, etc. [5,6,12,47]. Nonetheless, new targets are desirable. As an emerging virulence factor that plays a central role in biofilm development and nutrient acquisition, PaAP is an attractive new therapeutic target. Initial studies towards this end are promising, showing that common inhibitors of aminopeptidases, such as amastatin and Balsalazide inhibit biofilm formation in *P. aeruginosa* [37]. Furthermore, specific inhibitors of PaAP, designed based on certain elements in its 3D structure, slow down bacterial growth in protein-based culture media and block biofilm formation by *P. aeruginosa* [30]. These findings encourage further research on PaAP towards a better understanding of its biological roles, sites of action during infection, interactions with other proteins, and the relevant underlying mechanisms. Worth mentioning in this regard is that other bacterial metallo-aminopeptidases have also been proposed as potential therapeutic targets [48].

## 7. Conclusions and Future Directions

This review summarizes the current knowledge on PaAP, including structure, activation mechanism, cleavage specificity, biochemical properties, and regulation. PaAP is emerging as an important virulence factor, considered a promising new therapeutic target. In support of this, several inhibitors have been shown to slow down bacterial growth and block biofilm formation [30,37]. This, however, is only the first step on the path to success. Additional specific and potent inhibitors are needed. Besides active site-directed inhibitors, it is not unlikely that molecules targeting the PA domain could be inhibitory because the PA domain of other proteases is known to be involved in substrate recruitment and binding [49,50], and this could be true also for PaAP. The key to the interactions of PaAP with OMVs is also not clear, and the identification of relevant target proteins and binding sites could lay the ground for the design of new inhibitors. Elucidation of the structure–function relationships of the PA domain in mature PaAP is, therefore, an important research direction. Another challenge is that most inhibitors of virulence factors do not kill the bacteria [48]. Thus, it is important not only to identify new inhibitors, but also to elaborate new treatment protocols combining, for instance, antibiotics and PaAP inhibitors, or combining PaAP inhibitors with inhibitors of other virulence factors of *P. aeruginosa*. Furthermore, the leading virulence factors may vary in different infection stages or different types of infection, so optional new treatment approaches should be tested in different experimental models of *P. aeruginosa* infections. While this is a long-term goal, the ever-growing emergence of antibiotic-resistant strains and the constant need for new therapies both call for further research on PaAP as outlined above and beyond.

## Figures and Tables

**Table 1:** Cleavage specificity of PaAP.

**A.** Kinetic constants for hydrolysis of several amino acid p-NA derivatives													
pNA		*K_m_*					*K_cat_*				*K_cat_*/*K_m_*		
Substrate													
		(mM)					(s^−1^)				(s^−1^ mM^−1^)		
Leu	2.3	7.5	9.41		2.7	11.5	18.7		183.3	5	2.5		68.7
Ala	0.9	ND	ND		6.5	0.53	ND		17.4	0.59	ND		2.7
Met	1.9	4.7	ND		ND	0.96	3.7		ND	0.5	0.8		ND
Arg	0.9	3.9	ND		3	1.12	2.7		23.6	1.24	0.7		7.9
Lys	1.5	1.2	2.32		ND	4.1	6.9		ND	2.73	5.8		ND
**B.** Relative hydrolysis rates of the same amino acid p-NA derivatives as in **A**													
pNA								Relative Activity					
Substrate													
								(%)					
Leu				100				100				100	
Ala				3				ND				10	
Met				14				ND				ND	
Arg				ND				45				23.3	
Lys				ND				370				ND	

A: Values in red, green, black, and blue are from references [30,32,33,38], respectively. For a valid comparison, the original *K_cat_* and *K_cat_*/*K_m_* values in [30], which were expressed in min^−1^, were converted here to s^−1^ as a common denominator. *K_cat_* and *K_cat_*/*K_m_* values for Leu-pNA cleavage are all far higher than those found for Ala, Met, and Arg-pNA cleavage, i.e., Leu-pNA is a better substrate than Ala, Met, or Arg-pNA. *K_cat_*/*K_m_* values for Lys-pNA cleavage are either two-fold higher (green, ref. [33]) or about two-fold lower (red, ref. [30]) than those of Leu-pNA cleavage, indicating that Lys-pNA is a very good substrate for PaAP. ND, Not determined. B: Left to Right: Data are from references [17,32,39], respectively. The hydrolysis rate of Leu-pNA is considered 100%. ND, Not determined. The data are consistent with the kinetic parameters presented in part **A**, indicating that Leu-pNA is cleaved more rapidly than Ala, Met, and Arg-pNA, and that, as was found by Axelrad et al. [33] (see also Table 1A above), Lys-pNA is cleaved far more rapidly than Leu-pNA.

## References

[1] Strateva T., Mitov I. (2011). Contribution of an arsenal of virulence factors to pathogenesis of *Pseudomonas aeruginosa* infections. Ann. Microbiol..

[2] Diggle S.P., Whitely M. (2020). Microbe Profile: *Pseudomonas aeruginosa*: Opportunistic pathogen and lab rat. Microbiology.

[3] Jurado-Martin I., Sainz-Mejias M., McClean S. (2021). *Pseudomonas aeruginosa*: An audacious pathogen with an adaptable arsenal of virulence factors. Int. J. Mol. Sci..

[4] Behzadi P., Baráth Z., Gajdács M. (2021). It’s not easy being green: A narrative review on the microbiology, virulence and therapeutic prospects of multidrug-resistant *Pseudomonas aeruginosa*. Antibiotics.

[5] Liao C., Huang X., Wang Q., Yao D., Lu W. (2022). Virulence factors of *Pseudomonas aeruginosa* and antivirulence strategies to combat its drug resistance. Front. Cell. Infect. Microbiol..

[6] Qin S., Xiao X., Zhou C., Deng X., Lan L., Liang H., Song X., Wu M. (2022). *Pseudomonas aeruginosa* pathogenesis, virulence factors, antibiotic resistance, interaction with host, technology advances and emerging therapeutics. Signal Transduct. Targeted Ther..

[7] Barrett A.J. (2000). Proteases. Curr. Protoc. Prot. Sci..

[8] Hartley B.S. (1960). Proteolytic enzymes. Annu. Rev. Biochem..

[9] Kessler E., Safrin M. (2014). Elastinolytic and proteolytic enzymes. Pseudomonas Methods Protoc. Methods Mol. Biol..

[10] Galdino A.C.M., Branquinha M.H., Santos A.L.S., Viganor L., Charaborti S., Dhalla N.S. (2017). Pseudomonas aeruginosa and its arsenal of proteases: Weapons to battle the host. Pathophysiological Aspects of Proteases.

[11] Kessler E., Ohman D.E., Rawlings N.D., Salversen G.S. (2013). Pseudolysin. Handbook of Proteolytic Enzymes.

[12] Everett M.J., Davis D.T. (2021). *Pseudomonas aeruginsa* elastase (LasB) as a therapeutic target. Drug Discov. Today.

[13] Honek J.F., Rawlings N.D., Salversen G.S. (2013). Aeruginolysin. Handbook of Proteolytic Enzymes.

[14] Kessler E., Ohman D.E., Rawlings N.D., Salversen G.S. (2013). Staphylolysin. Handbook of Proteolytic Enzymes.

[15] Eliott B.W., Cohen C. (1996). Isolation and characterization of a lysine-specific protease from *Pseudomonas aeruginosa*. J. Biol. Chem..

[16] Malloy J.L., Veldhuizen R.A.W., Thibodeux B.A., O’Callaghan R.J., Wright J.R. (2005). *Pseudomonas aeruginosa* protease IV degrades surfactant proteins and inhibits surfactant host defense and biophysical functions. Am. J. Physiol. Lung Cell. Mol. Physiol..

[17] Cahan R., Axelrad I., Safrin M., Ohman D.E., Kessler E. (2001). A secreted aminopeptidase of *Pseudomonas aeruginosa*: Identification, primary structure, and relationship to other aminopeptidases. J. Biol. Chem..

[18] Bleve S., Viarre V., Salacha R., Michel G.P.F., Filloux A., Voulhoux R. (2010). Protein secretion systems in *Pseudomonas aeruginosa*: A wealth of pathogenic weapons. Int. J. Med. Microbiol..

[19] Chandha J., Harjai K., Chhibber S. (2022). Revisiting the virulence hallmarks of *Pseudomonas aeruginosa*: A chronicle through the perspective of quorum sensing. Environ. Microbiol..

[20] Toyofuku M., Roschitzki B., Riedel K., Eberl L. (2012). Identification of proteins associated with the *Pseudomonas aeruginosa* biofilm extracellular matrix. J. Proteome Res..

[21] Zhao T., Zhang Y., Wu H., Wang D., Chen Y., Zhu M.J., Ma L.Z. (2018). Extracellular aminopeptidase modulates biofilm development of *Pseudomonas aeruginosa* by affecting matrix exopolysaccharide and bacterial cell death. Environ. Microbiol. Rep..

[22] Esoda C.N., Kuehn M.J. (2019). *Pseudomonas aeruginosa* leucine aminopeptidase influences early biofilm composition and structure via vesicle-associated antibiofilm activity. mBio.

[23] Braun P., De Groot A., Bitter W., Tommassen J. (1998). Secretion of elastinolytic enzymes and their propeptides by *Pseudomonas aeruginosa*. J. Bacteriol..

[24] Greenblatt H.M., Almog O., Maras B., Spungin-Bialik A., Barra D., Blumberg S., Shoham G. (1997). *Streptomyces griseus* aminopeptidase: X-ray crystallographic structure at 1.75 Å resolution. J. Mol. Biol..

[25] Gao X., Cui W., Tian Y., Zhou Z. (2013). Over-expression, secretion, biochemical characterisation, and structure analysis of *Bacillus subtilis* aminopeptidase. J. Sci. Food Agric..

[26] Chevrier B., D’Orchymont H., Schalk C., Tarnus C., Moras D. (1996). The structure of the *Aeromonas proteolytica* aminopeptidase complexed with a hydroxamate inhibitor. Involvement in catalysis of Glu151 and two zinc ions of the co-catalytic unit. Eur. J. Biochem..

[27] Robinson T., Smith P., Alberts E.R., Colussi-Pelaez M., Schuster M. (2020). Cooperation and cheating through a secreted aminopeptidase in the *Pseudomonas aeruginosa* RpoS response. mBio.

[28] Bauman S.J., Kuehn M.J. (2006). Purification of outer membrane vesicles from *Pseudomonas aeruginosa* and their activation of an IL-8 response. Microbes Infect..

[29] Bauman S.J., Kuehn M.J. (2009). *Pseudomonas aeruginosa* vesicles associate with and are internalized by human lung epithelial cells. BMC Microbiol..

[30] Harding C.J., Bischoff M., Bergkessel M., Czekster C.M. (2023). An anti-biofilm cyclic peptide targets a secreted aminopeptidase from *P. aeruginosa*. Nat. Chem. Biol..

[31] Sarnovsky R., Rea J., Makowski M., Hertle R., Kelly C., Antignani A., Pastrana D.V., FitzGerald D.J. (2009). Proteolytic cleavage of a C-terminal prosequence, leading to autoprocessing at the N Terminus, activates leucine aminopeptidase from *Pseudomonas aeruginosa*. J. Biol. Chem..

[32] Wu Y.T., Zhou N.D., Zhou Z.M., Gao X.X., Tian Y.P. (2014). A thermo-stable lysine aminopeptidase from *Pseudomonas aeruginosa*: Isolation, purification, characterization, and sequence analysis. J. Basic Microbiol..

[33] Axelrad I., Safrin M., Cahan R., Suh S.-J., Ohman D.E., Kessler E. (2021). Extracellular proteolytic activation of *Pseudomonas aeruginosa* aminopeptidase (PaAP) and insight into the role of its non-catalytic N-terminal domain. PLoS ONE.

[34] Gaur R., Grover T., Sharma R., Kapoor S., Khare S.K. (2010). Purification and characterization of a solvent stable aminopeptidase from *Pseudomonas aeruginosa*: Cloning and analysis of aminopeptidase gene conferring solvent stability. Proc. Biochem..

[35] Nouwens A.S., Beatson S.A., Whitchurch C.B., Walsh B.J., Schweizer H.P., Mattick J.S., Cordwell S.J. (2003). Proteome analysis of extracellular proteins regulated by the las and rhl quorum sensing systems in *Pseudomonas aeruginosa* PAO1. Microbiology.

[36] Gonzales T., Robert-Baudouy J. (1996). Bacterial aminopeptidases: Properties and function. FEMS Microbiol. Rev..

[37] Zhao T., Zhang J., Tang M., Ma L.Z., Lei X. (2019). Development of an effective fluorescence probe for discovery of aminopeptidase inhibitors to suppress biofilm formation. J. Antibiot..

[38] Tang A.-X., Liu H., Liu Y.-Y., Li Q.-Y., Qing Y.-M. (2017). Purification and characterization of a novel β-Cypermethrin-degrading aminopeptidase from *Pseudomonas aeruginosa* GF31. J. Agric. Food Chem..

[39] Pei X.-D., Li F., Yue S.-Y., Huang X.-N., Gao T.-T., Jiao D.-Q., Wang C.-H. (2023). Production and characterization of novel thermos- and organic solvent-stable keratinase and aminopeptidase from *Pseudomonas aeruginosa* 4-3 for effective poultry feather degradation. Environ. Sci. Pollut. Res..

[40] Mahon P., Bateman A. (2000). The PA domain: A protease-associated domain. Protein Sci..

[41] Luo X., Hofmann K. (2001). The protease-associated domain: A homology domain associated with multiple classes of proteases. Trends Biochem. Sci..

[42] Oh J., Li X.-H., Kim S.-K., Lee J.-H. (2017). Post-secretional activation of protease IV by quorum sensing in *Pseudomonas aeruginosa*. Sci. Rep..

[43] Schuster M., Lostroh P.C., Ogi T., Greenberg E.P. (2003). Identification, timing, and signal specificity of *Pseudomonas aeruginosa* quorum-controlled genes: A transcriptome analysis. J. Bacteriol..

[44] Schuster M., Hawkins A.C., Harwood C.S., Greenberg E.P. (2004). The *Pseudomonas aeruginosa* RpoS regulon and its relationship to quorum sensing. Mol. Microbiol..

[45] Ramirez-Larrota J.S., Eckhard U. (2022). An introduction to bacterial biofilms and their proteases, and their role in host infection and immune evasion. Biomolecules.

[46] Franklin M.J., Nivens D.E., Weadge J.T., Howell L.P. (2011). Biosynthesis of the *Pseudomonas aeruginosa* extracellular polysaccharides, Alginate, Pel, and Psl. Front. Microbiol..

[47] Dolan S.K. (2020). Current knowledge and future directions in developing strategies to combat *Pseudomonas aeruginosa* infection. J. Mol. Biol..

[48] González-Bacerio J., Varela A.C., Aguado M.E., Izquierdo M., Méndez Y., del Rivero M.A., Rivera D.G. (2022). Bacterial metalo-aminopeptidases as targets in human infectious diseases. Curr. Drug Targets.

[49] Li H.J., Tang B.L., Shao X., Liu B.X., Zheng X.Y., Han X.X., Li P.Y., Zhang X.Y., Song X.Y., Chen X.L. (2016). Characterization of a new S8 serine protease from marine sedimentary photobacterium sp. A5-7 and the function of its protease-associated domain. Front. Microbiol..

[50] McKenna S., Aylward F., Miliara X., Lau R.J., Huemer C.B., Giblin S.P., Huse K.K., Liang M., Reeves L., Pearson M. (2023). The protease associated (PA) domain in ScpA from *Streptococcus pyogenes* plays a role in substrate recruitment. Biochim. Biophys. Acta Proteins Proteom..

